# Antimicrobial susceptibility profiles of human and piglet *Clostridium difficile* PCR-ribotype 078

**DOI:** 10.1186/2047-2994-2-14

**Published:** 2013-04-08

**Authors:** Elisabeth C Keessen, Marjolein PM Hensgens, Patrizia Spigaglia, Fabrizio Barbanti, Ingrid MJG Sanders, Ed J Kuijper, Len JA Lipman

**Affiliations:** 1 Department of Medical Microbiology, Leiden University Medical Center, PO Box 9600, Leiden 2300 RC, the Netherlands; 2 Institute for Risk Assessment Sciences, Utrecht University, PO Box 80175, Utrecht 3508 TD, the Netherlands; 3 Department of Infectious, Parasitic and Immune-mediated Diseases, Instituto Superiore di Sanita’, Rome, Italy

## Abstract

In the last decade, outbreaks of nosocomial *Clostridium difficile* infections (CDI) occurred worldwide. A new emerging type, PCR-ribotype 027, was the associated pathogen. Antimicrobial susceptibility profiles of this type were extensively investigated and used to partly explain its spread. In Europe, the incidence of *C. difficile* PCR-ribotype 078 recently increased in humans and piglets. Using recommendations of the European Committee on Antimicrobial Susceptibility Testing (EUCAST) and the Clinical and Laboratory Standards Institute (CLSI) we studied the antimicrobial susceptibility to eight antimicrobials, mechanisms of resistance and the relation with previously prescribed antimicrobials in human (n=49) and porcine (n=50) type 078 isolates. Human and porcine type 078 isolates showed similar antimicrobial susceptibility patterns for the antimicrobials tested. In total, 37% of the isolates were resistant to four or more antimicrobial agents. The majority of the human and porcine isolates were susceptible to amoxicillin (100%), tetracycline (100%) and clindamycin (96%) and resistant to ciprofloxacin (96%). More variation was found for resistance patterns to erythromycin (76% in human and 59% in porcine isolates), imipenem (29% in human and 50% in porcine isolates) and moxifloxacin (16% for both human and porcine isolates). MIC values of cefuroxim were high (MICs >256 mg/L) in 96% of the isolates. Resistance to moxifloxacin and clindamycin was associated with a *gyr*(A) mutation and the presence of the *erm*(B) gene, respectively. A large proportion (96%) of the erythromycin resistant isolates did not carry the *erm*(B) gene. The use of ciprofloxacin (humans) and enrofloxacin (pigs) was significantly associated with isolation of moxifloxacin resistant isolates. Increased fluoroquinolone use could have contributed to the spread of *C. difficile* type 078.

## Introduction

*Clostridium difficile* is a ubiquitous organism that recently emerged in both humans and animals. In humans, *C. difficile* is the leading cause of nosocomial diarrhea. In the last decade incidences of *C. difficile* infections (CDI) increased, which was partly explained by the emergence of the hypervirulent *C. difficile* PCR-ribotype 027 [1-3]. Since 2006, the incidence of nosocomial CDI is constant in The Netherlands, encompassing a decrease of CDI caused by type 027 [4]. Meanwhile, *C. difficile* PCR-ribotype 078 increased and it became the third most commonly type found in human infections in The Netherlands and Europe [4-6]. Type 078 causes severe diarrhea in 40% of the patients and is associated with CDI-related mortality in 4 percent after 30 days [6]. Patients infected with type 078 are younger and the disease more frequently occurs outside healthcare facilities [6]. Besides a high prevalence in human CDI, type 078 is the main cause for CDI in neonatal piglets [7-9]. In the United States, CDI is even the most commonly diagnosed cause of enteritis in neonatal piglets [10]. Morbidity in an infected farrowing facility is on average 2/3 of litters and 1/3 of individual piglets [11], but may be as high as 97-100% [10]. Mortality due to *C. difficile* is usually low, nonetheless, outbreaks with mortality rates of 16% have been reported [12].

In humans, the use of antimicrobials, in particular cephalosporins, clindamycin and fluoroquinolones, is a major risk factor for CDI [13]. Resistance to newer fluoroquinolones was used to partly explain the emergence of type 027 in healthcare centers [1-3]. For type 078, especially porcine type 078, antimicrobial susceptibility profiles are less extensively investigated. Therefore, the goal of this study is to analyze susceptibility profiles and mechanisms of resistance for *C. difficile* type 078 isolates of human and porcine origin and to assess if a specific resistance is related to prior antimicrobial exposure.

## Materials and methods

### Sample selection

Between April 2009 and January 2010 we visited 25 Dutch pig breeder farms. Twenty-two farms had problems with recurrent diarrhea in neonatal piglets, three farms had not. At farms where diarrhea was present only diarrheal piglets were sampled; at farms without diarrhea, piglets without signs of disease were sampled. At every farm, faecal samples were taken from piglets from at least three different litters. In total, 262 porcine faecal samples were cultured as described previously [9]. DNA was isolated from single *C. difficile* colonies using the QIA amp DNA mini blood kit (QIAgen) according manufacturer’s protocol. All isolates were confirmed as *C. difficile* by an in-house developed PCR specific for *C. difficile* glutamate dehydrogenase gene (GluD) and typed as previously described by Bidet et al. [7] and Paltansing et al. [23]. Out of 139 type 078 isolates, per farm two *C. difficile* type 078 isolates were selected (random), except for one farm where three isolates were selected and one farm where one isolate was selected. Piglets were one to seven days old. Forty-nine human type 078 isolates were selected from all samples submitted to the Dutch national reference laboratory (mainly nosocomial diarrhea) between June 2006 and May 2009. To avoid enhanced selection of epidemic strains, only one strain per month per hospital was included.

### Antimicrobial susceptibility and mechanisms of resistance

*C. difficile* was cultured in Brain Heart Infusion (BHI) broth for 24 hours at 37°C in anaerobic conditions. Subsequently, cultures were diluted to a 1.0 McFarland standard and swabbed on Brucella blood agar plates, supplemented with haemin 5 mg/L and vitamin K1 1 mg/L, E-test strips (AB BioMérieux) were applied for tetracycline, amoxicillin, erythromycin, clindamycin, moxifloxacin, imipemen, cefuroxim, and ciprofloxacin. Minimal inhibitory concentrations (MICs) were determined after 48 hours incubation. Antimicrobial susceptibility patterns of human and piglet origin were compared to antimicrobial susceptibility patterns of wild-type *C. difficile* isolates, as recently determined by the European Committee on Antimicrobial Susceptibility Testing (EUCAST) [14]. To classify strains as resistant or susceptible, Clinical and Laboratory Standards Institute (CLSI) breakpoints were used [15].

The mechanisms of resistance to tetracycline, erythromycin, clindamycin and moxifloxacin were investigated in more detail. Tetracycline isolates with MIC ≥8 mg/L were tested for the presence of *tet*(M), *int* and *tnd*X genes according to previous publications by Spigaglia et al. [16], [17]. The last two genes were used as markers for Tn*916* and Tn*5397*-related elements, respectively. Erythromycin and clindamycin isolates with a MIC ≥4 mg/L were tested for the presence of the *erm*(B) gene and moxifloxacin isolates with an MIC ≥4 mg/L were tested for mutations in the *gyr*(A) and *gyr*(B) genes according to previously described methods by Spigaglia et al. [18,19].

### Clinical data collection and statistical analysis

At every farm the use of antimicrobials of the sampled piglets was noted since their birth. Information on the antibiotic treatment of the patients in the three months prior to the start of the diarrhea was collected at time of diagnosis. This was done by contacting the physician in charge and consulting patient records. Data were processed using SPAW statistical software for Windows, version 17.0. For comparison of binary data, the Chi-square test was used. If the expected cell count in the contingency table was less than five, the Fisher’s exact test was used.

## Results

*C. difficile* type 078 was detected at all 25 farms (with and without diarrhoeal problems). Fifty porcine 078 isolates and 49 human isolates were selected and included in the study.

### Antimicrobial susceptibility and mechanisms of resistance

**Figure 1:**
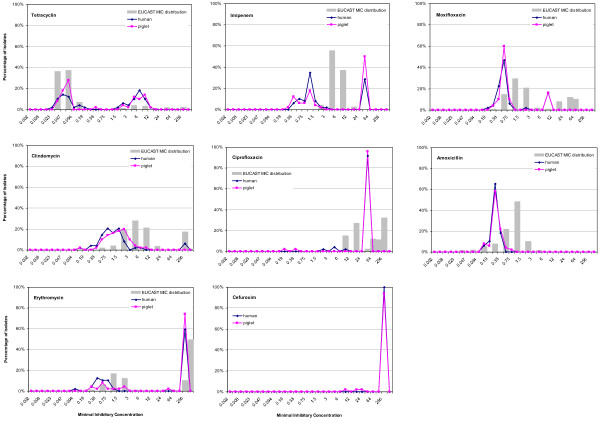
**Antimicrobial susceptibility patterns of piglet and human *C. difficile *for eight antimicrobial agents, the grey bars indicate the antimicrobial susceptibility patterns of wild-type *C. difficile *as determined by EUCAST **[14]**.**

Both human and porcine isolates had susceptibility patterns comparable to other PCR-ribotypes in Europe (grey bars in Figure 1). As shown in Table 1, MIC_90_ values were also equal or lower than found in the EUCAST MIC distribution and similar antimicrobial susceptibility patterns were observed in human and porcine type 078 isolates for the antimicrobials tested. The majority of isolates were susceptible to amoxicillin (100%), tetracycline (100%) and clindamycin (96%). Interestingly, only 16% of the isolates were resistant to moxifloxacin. Variable resistance percentage was observed for erythromycin (76% in human and 59% in porcine isolates) and imipenem (29% in human and 50% in porcine isolates). High MIC values were observed for ciprofloxacin (94% resistant) and cefuroxim. In total 14 human and 18 porcine isolates were resistant to four antimicrobials, while 5 porcine isolates were resistant to five antimicrobials (data not shown).

**Table 1:** Antibiotic resistance against eight antimicrobial agents, stratified for origin of the sample

** Antibiotic agent**	**MIC **_**90 **_**(mg/L)**			**Resistant isolates according to CLSI**^**b **^**(%)**			
	**Human n=49**	**Pig n=50**	**ECOFF**^**a**^**(mg/L)**	**Breakpoint (mg/L)**	**Human n=49**	**Pig n=50**	**Difference p-value**
Amoxicillin	0.25	0.25	2	16	0 (0%)	0 (0%)	1.00
Cefuroxim	≥256	≥256	-	-	NA	NA	NA
Clindamycin	2	2	≥256	8	3 (6%)	1 (2%)	0.30
Erythromycin	≥256	≥256	≥256	8	29 (59%)	38 (76%)	0.07
Ciprofloxacin	≥32	≥32	≥32	8	46 (94%)	48 (96%)	0.044
Moxifloxacin	8	8	≥64	8	8 (16%)	8 (16%)	0.067
Tetracycline	8	8	8	16	0 (0%)	0 (0%)	1.00
Imipenem	≥32	≥32	8	16	14 (29%)	25 (50%)	0.03

^a^ ECOFF: Epidemiological cut-off value - The European Committee on Antimicrobial Susceptibility Testing (EUCAST).

^b^ CLSI: Clinical and Laboratory Standards Institute.

NA: not applicable. This is stated because no breakpoint was determined by the CLSI and, therefore, the percentage of resistant isolates could not be determined.

Of the 67 isolates resistant to erythromycin only four isolates (6%) were resistant to clindamycin, whereas all clindamycin resistant isolates were resistant to erythromycin (data not shown). The 16 isolates resistant to moxifloxacin were also resistant to ciprofloxacin (data not shown).

Six human isolates and eight porcine isolates with MIC values ranging from 8 to 12 mg/L and considered susceptible, since the breakpoint for tetracycline is ≥16 mg/L, contained a *tet*(M) gene and a Tn*916*-like element (Table 2). Among the isolates resistant to erythromycin, only three from human (MIC ≥ 256 mg/L) had an *erm*(B) gene. These isolates were also high-level resistant to clindamycin (MIC ≥ 256 mg/L). The *erm*(B) gene was not found in a porcine isolate with resistance to both erythromycin and clindamycin. Four of the seven porcine isolates (57%; one porcine isolate was not tested) and all the human isolates (100%) resistant to moxifloxacin had the amino acid substitution Thr82 to Ile in GyrA.

**Table 2:** Mechanisms of resistance found in C. difficile type 078 isolates

** Antibiotic**	**Origin of isolate **^**a **^**(total number)**	** Mechanism of resistance**	**N. of positive isolates (%)**
Tetracycline	Human (6)	*tet*(M)	6 (100%)
	Porcine (8)		8 (100%)
Erythromycin	Human (29)	*erm*(B)	3 (10%)
	Porcine (38)		0 (0%)
Clindamycin	Human (3)	*erm*(B)	3 (100%)
	Porcine (1)		0 (0%)
Moxifloxacin	Human (8)	Aminoacid substitution in GyrA	8 (100%)
	Porcine (7)		4 (57%)

^a^: All isolates with tetracycline MIC ≥8 mg/L (n=14), erythromycin MIC ≥4 mg/L (n=67) or clindamycin MIC ≥4 mg/L (n=4) were analysed for resistance mechanisms. Seven of eight porcine isolates with moxifloxacin MIC ≥4 mg/L were tested for mutations.

### Human and farm specific antimicrobial use

Human antimicrobial use was known for 34 of 50 patients (68%). The most frequently used antimicrobial classes were: penicillins (18/34; 53%), cephalosporins (17/34; 50%) and fluoroquinolones (9/34; 26%; mainly ciprofloxacin). As all type 078 isolates had low MIC values for amoxicillin and high MIC values for the cephalosporin cefuroxim, we only investigated the concomitant use of quinolones and the resistance to moxifloxacin. The use of fluoroquinolones (mainly ciprofloxacin) was significantly associated with resistance to moxifloxacin (4/5 patients with prior fluoroquinolone use were resistant, versus 0/24 without fluoroquinolone treatment; p<0.01). The use of fluoroquinolones was not associated with resistance to ciprofloxacin, as virtually all isolates (94%) were resistant.

Penicillins (7/25 farms), the 3^rd^ generation fluoroquinolone enrofloxacin (6/25 farms) and colistine (4/25 farms) were commonly used in piglets in this study. At the three farms with no history of diarrhea, no antimicrobials were given to the sampled piglets. Similar to humans, the use of enrofloxacin was significantly associated with resistance to moxifloxacin, which was found in 5 of the 12 piglets treated with enrofloxaxin, and in 3 of the 36 piglets not treated with a fluorquinolone (p= 0.017). One farm was excluded from this analysis because no information on the use of antimicrobials was available for this farm.

## Discussion

We tested *C. difficile* type 078 isolates from humans and piglets for their minimal inhibitory concentration against eight antimicrobial agents and the results were evaluated using the wild type distribution data of EUCAST and the breakpoint values recommended by CLSI. In general, the results obtained indicate that type 078 isolates have similar antimicrobial susceptibility profiles as other PCR-ribotypes, though moxifloxacin resistance was found somewhat less in the examined strains, since only 16% of them were resistant to this antibiotic. Thirty seven percent of the isolates were resistant to four or more antimicrobials, a percentage comparable to those already described for other *C. difficile* types [20].

A study concerning type specific risk factors for human CDI in The Netherlands found that prior use of fluoroquinolones (mainly ciprofloxacin) was associated with CDI due type 078 [6]. Of type 078 isolates included is this study, 94% was resistant to ciprofloxacin (second generation fluoroquinolone), but moxifloxacin (third generation) resistance was low, which is in concordance with the low resistance (27%) found by Salomon et al., (2011). During the rapid emergence of type 027 a decade ago, resistance of type 027 against moxifloxacin was frequent [21], which was also in line with the association of type 027 and fluoroquinolones in epidemiological studies. Moxifloxacin is not among the most frequently used human fluoroquinolones and is not available for veterinary use. It was however possible to establish an epidemiological link between resistance to moxifloxacin and the administration of fluoroquinolones in general (moxifloxacin and others) in human patients (p<0.01) and piglets (p=0.02). Since resistance can be linked with treatment with fluoroquinolones, but resistance is infrequent compared to other PCR-ribotypes isolated from human disease (Figure 1), we doubt that increased fluoroquinolone use alone explains the recent emergence of *C. difficile* type 078 in humans. In piglets, however, type 078 is virtually the only PCR ribotype that causes disease, indicating that a species border exists. The resistance against fluoroquinolones could have contributed to emergence of Type 078 in pigs after the introduction of type 078 in pig farms and the subsequent spread of type 078, as fluoroquinolone use was present in many pig farms and no other *C. difficile* PCR ribotypes were found.

In the Netherlands, there is a remarkable difference in antimicrobial use in humans versus animals. The use of antimicrobials in human medicine in the Netherlands is among the lowest in the European Union, while veterinary use of antimicrobials is among the highest [22,23]. The most frequent used antibiotics for all types of disease in sows and suckling piglets are tetracyclines, followed by trimethoprim/sulfonamides (co-trimoxazole) and penicillins [24]. This difference in antimicrobial use was however not reflected in different resistance profiles in our study.

The only discrepancy in human and porcine antimicrobial resistance profile was found for imipenem, with resistance in 29% of the human isolates and in 50% of the porcine isolates. Since imipenem is not used in porcine medicine, a higher level of resistance to this antimicrobial in porcine isolates compared to human isolates was unexpected. In the United states where imipenem is neither used in porcine medicine 100% of piglet isolates had MIC values of ≥16 mg/L [25]. No explanation for this higher level of resistance can be found. Cross-resistance with other antibiotics has not been described for imipenem in gram-positive bacteria. Besides considerable overlap in susceptibility profiles, human and porcine 078 strains also had great genetic similarity when evaluated by multilocus variable-number tandem repeat analysis (MLVA), whole-genome analysis and pulsed-field gel electrophoresis (PFGE) [7,26-28]. Using these methods, numerous human and porcine type 078 isolates were indistinguishable and were therefore suggested to have a high-level of genetic relatedness. Together with the increasing incidence of type 078, the association with community-associated CDI and the presence of type 078 in more than 90% of the piglets with colonization with *C. difficile*, the hypothesis arose that human and piglet type 078 had a common origin. The results of our study contribute to this hypothesis since resistance patterns highly overlapped despite different antimicrobial pressure [29]. However, as many resistance genes are situated on transposons, antimicrobial susceptibility studies cannot give definitive insight in the (possible) common source of human and porcine CDI.

In this study, resistance to moxifloxacin was associated with the aminoacid substitution Thr82 to Ile in GyrA, as already observed in the majority of European *C. difficile* clinical isolates resistant to fluoroquinolones [19,30,31]. Surprisingly, no tetracycline resistant isolates were found in our study. However, MIC values between 8 and 12 mg/L were observed in less than 20% of the isolates analysed. It has been shown that *C. difficile* strains with reduced susceptibility to tetracycline often carry a *tet*(M) gene and are inducibly resistant when exposed to sub-inhibitory concentrations of this antibiotic [32]. A recent Irish study found that MLS_B_ resistance in type 078 is less frequently associated with *erm*(B) than other types [21]. Our results support this observation since only a minority of the erythromycin resistance (up to 10%) can be explained by the presence of an *erm*(B) gene. Anyway, we did not observe any clindamycin resistant, erythromycin susceptible 078 isolate, as reported in the study mentioned above. What causes the resistance to erythromycin in 078 isolates is currently unknown. Although other erm(B) negative anaerobes possess one of the other erm genes (frequently erm classes A, F or Q) or overexpress an efflux pump, these have not been determined in C. difficile type 078 [21].

The strengths of our study in comparison with other reports on antimicrobial susceptibility data of *C. difficile* type 078 are that a large number of isolates of different origin were included and eight different antimicrobials were tested [6,7,25,33]. Additionally, we compared the antimicrobial susceptibility data with previous antimicrobial treatments of patients and piglets. Limitations of the study are the lack of more detailed data on previous antimicrobial use (preferably expressed as daily defined dosages) and the fact that iinformation in piglets was obtained by interviewing the farmer, which enables reporting bias. Currently, attempts are underway to use defined daily dosage (DDD) as a measure for antimicrobial use, applicable for both humans and animals.

## Conclusion

The results of our study contribute to the hypothesis that human and piglet type 078 have a common origin, since similar antimicrobial susceptibility patterns were found for the antimicrobials tested in human and porcine type 078 isolates, despite a different antimicrobial pressure in humans and pigs. The use of fluoroquinolones was significantly associated with resistance to moxifloxacin in both human and porcine isolates. It is possible that after the introduction of *C. difficile* PCR-ribotype 078 in pig farms, fluoroquinolones contributed to its rapid spread.

## Competing interests

The authors declare that they have no competing interests.

## Authors’ contribution

EK, MH and IS preformed the antimicrobial resistance testing. EK and MH additionally collected the samples and the clinical data, furthermore, they wrote the first draft of the manuscript. FB and PS performed tests concerning the mechanism of resistance and revised drafts of the manuscript. LL and EK designed the study and revised drafts of the manuscript. All authors read and approved the final manuscript.
